# Testicular Rupture: Clinical, Sonographic, and Surgical Correlation in an Adolescent Patient

**DOI:** 10.7759/cureus.78688

**Published:** 2025-02-07

**Authors:** Zafeiria G Papathanassiou, Konstantina Michalopoulou, Vasileios Alexopoulos, Antonios Panagidis

**Affiliations:** 1 Radiology, Patras Children’s Hospital, Patras, GRC; 2 Pediatric Surgery, Patras Children’s Hospital, Patras, GRC

**Keywords:** blunt scrotal trauma, scrotal hematocele, scrotal ultrasound, surgical management of scrotal trauma, testicular hematoma, testicular rupture

## Abstract

Testicular tears, although uncommon, require early detection and control for symptom relief and, most importantly, organ salvage. Clinical examination of the scrotum cannot solely facilitate a valid diagnosis. Depending on the severity of the scrotal injury, treatment can be either conservative or surgical. Scrotal ultrasound is the preferred examination not only for the depiction and surveillance of testicular ruptures but also for guiding treatment planning. This case study describes the clinical assessment, imaging investigation, and management of an extensive testicular tear in a 14-year-old adolescent football athlete.

## Introduction

Scrotal injuries comprise less than 1% of all trauma cases and most commonly affect young males aged 10 to 30 years [1]. There is an increased risk of these injuries among young athletes [2]. The severity of blunt scrotal trauma ranges from minor tears to complete organ loss [3]. Although not lethal, the loss of a testicle can compromise fertility and negatively influence social behavior, especially among teenagers [2]. The term "testicular rupture" refers to a tear in the fibrous sheath of the testis, known as the tunica albuginea, resulting in the extrusion of seminiferous tubules into the scrotal sac [4]. Patients typically present with nonspecific symptoms such as acute scrotal pain and swelling, nausea, and sometimes vomiting [2]. Ultrasound is an effective tool for detecting and monitoring all types of testicular trauma, which physical examination may not accurately assess due to scrotal swelling and tenderness. Testicular ruptures are usually treated surgically, either through debridement or orchiectomy [1,2,4,5]. Despite initial skepticism, a more conservative therapeutic approach has been selectively advocated and established in recent years [1,4,5]. This case highlights the significance of ultrasound findings in early diagnosis and re-evaluates the importance of an urgent surgical approach.

## Case presentation

A 14-year-old football player was admitted nearly eight hours after being kicked in the genitals, with worsening pain and persistent swelling of the right hemiscrotum. On physical exam, an ecchymosis and enlargement of the right hemiscrotum were depicted. The right testis was difficult to examine. Accompanying symptoms included nausea and vomiting during palpation. Cremaster reflex was present on both sides. No hematuria or abdominal symptoms were noted.

**Figure 1:**
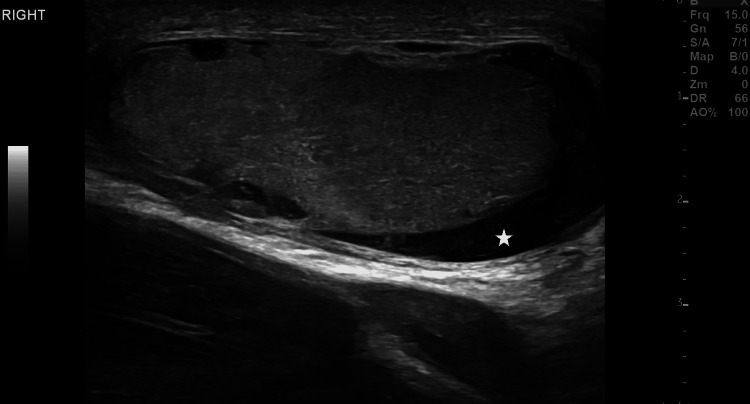
Testicular hematocele. A longitudinal gray-scale ultrasound image revealed the presence of echogenic fluid (asterisk) in the right hemiscrotum, suggestive of a hematocele. Additionally, there was scrotal wall thickening.

**Figure 2:**
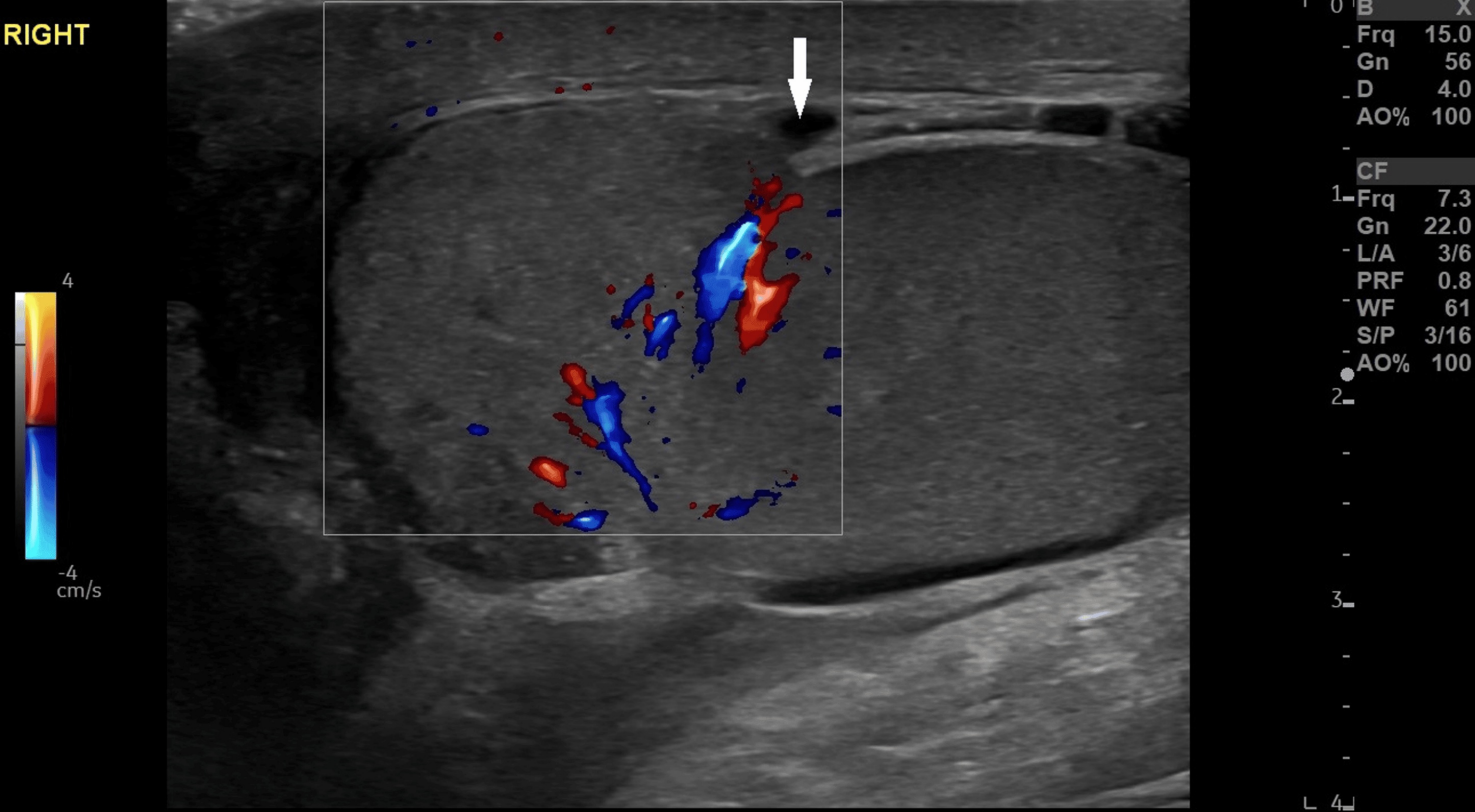
Testicular rupture. Gray-scale and color Doppler image of the right testis exhibited focal discontinuity of the tunica albuginea (arrow) in association with heterogeneous testicular echotexture.

**Figure 3:**
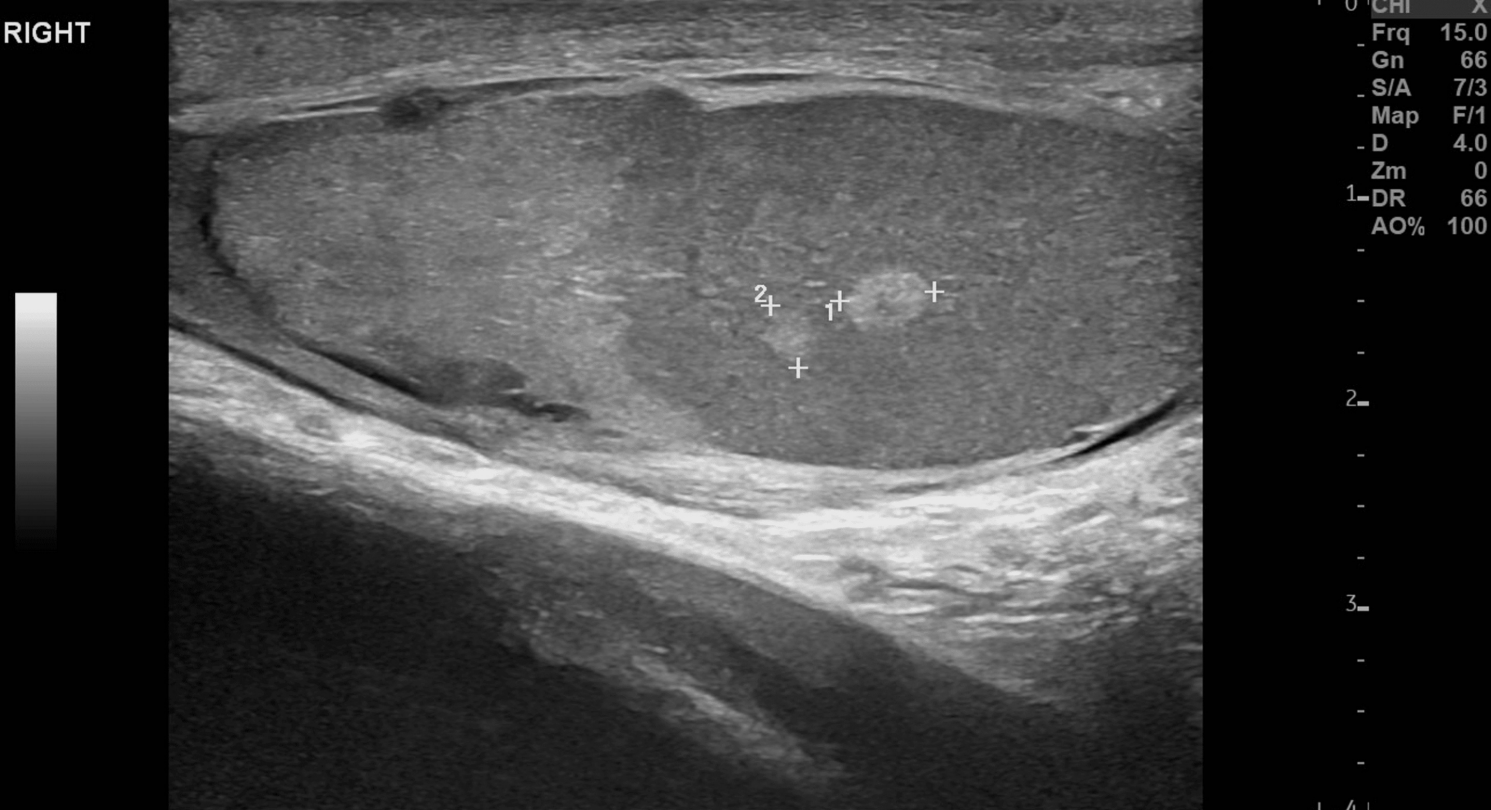
Focal testicular lesions. Small-sized, scattered, intraparenchymal, echogenic testicular areas (between cursors), indicative of focal hematomas.

**Figure 4:**
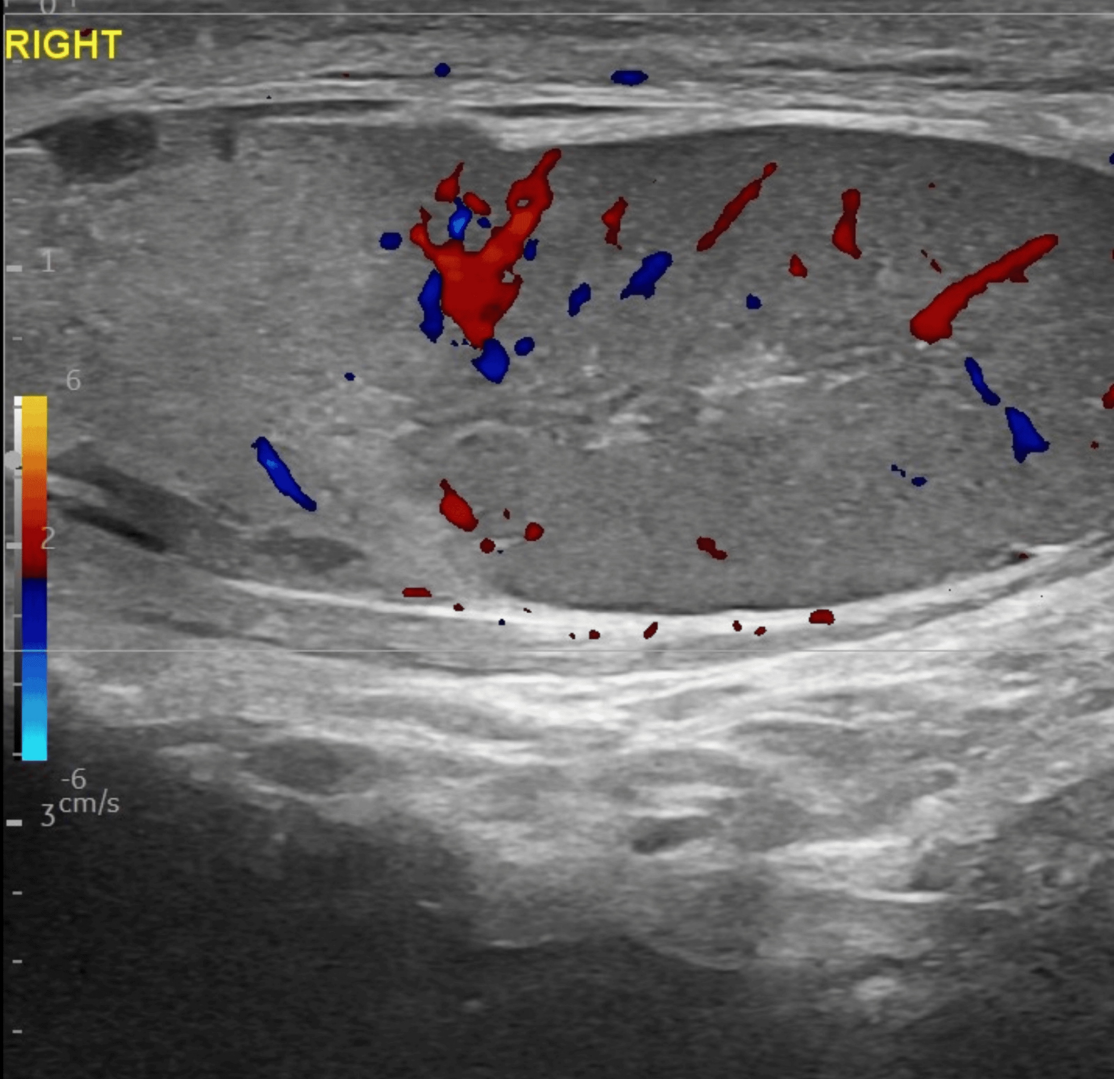
Testicular heterogeneity. The sagittal ultrasound color Doppler plane demonstrated a heterogeneous and avascular echotexture of the upper pole of the right testis in the setting of a larger testicular hematoma contusion.

Grayscale ultrasonography using a linear 12 MHz probe revealed an enlarged right testicle surrounded by echogenic fluid; a finding suggestive of hematocele (Figure 1). Other remarkable imaging findings included scrotal wall thickening and diffusely distorted parenchymal echotexture, especially at the proximal pole of the testis. The hyperechoic linear border of the testis indicative of the tunica albuginea was breached in the setting of a testicular tear (Figure 2). A few scattered, focal, hyperechoic, intraparenchymal areas represented small intratesticular hematomas (Figure 3). On color Doppler exam, a large echogenic area was revealed at the upper pole of the testis lacking vascularity (Figure 4). There was no evidence of testicular torsion.

The young patient was admitted to the surgery department and he was initially treated conservatively for approximately 36 hours with recumbency, scrotal support, IV antibiotics, and anti-inflammatory and analgesic agents.

**Figure 5:**
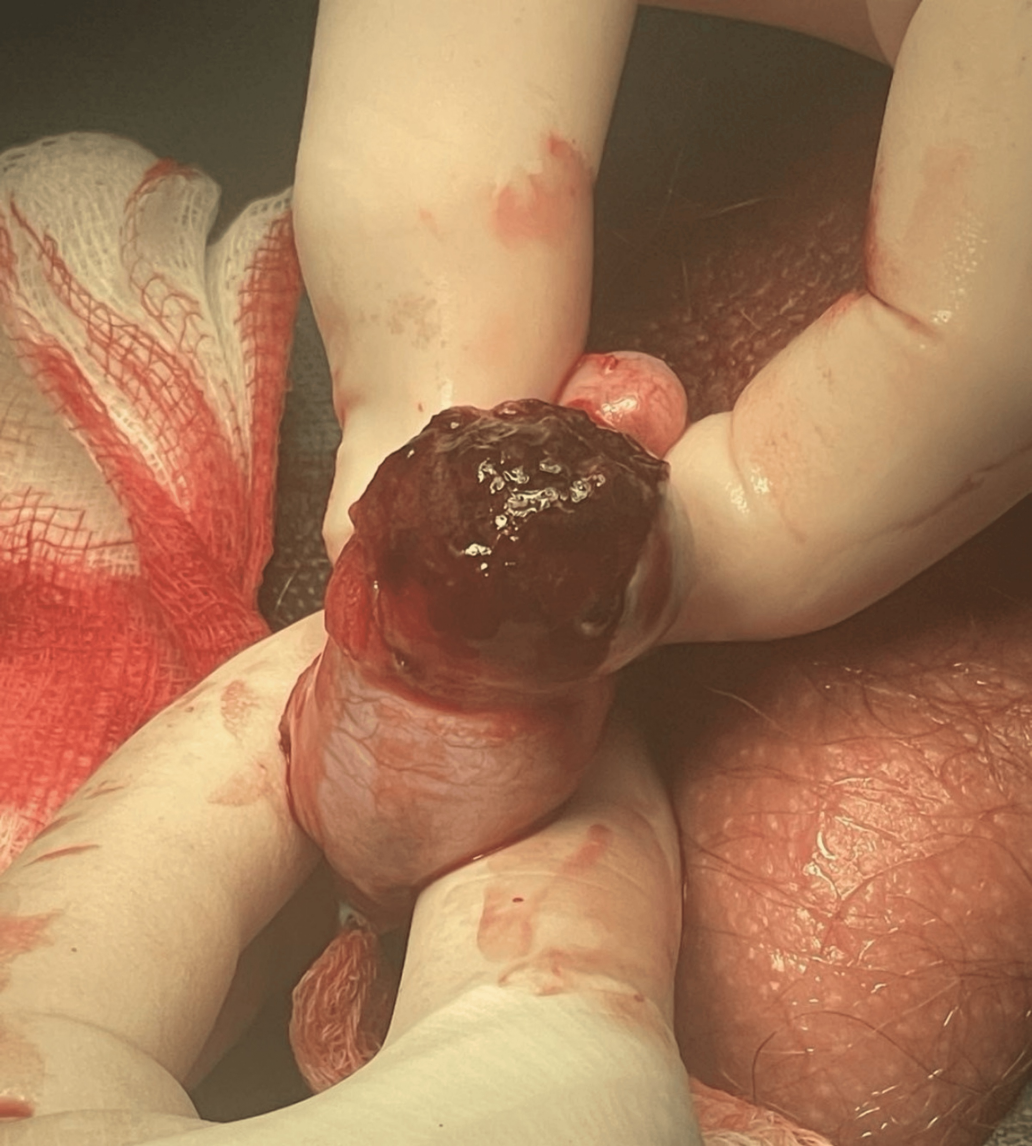
Release - debridement of necrotic testicular tissue.

**Figure 6:**
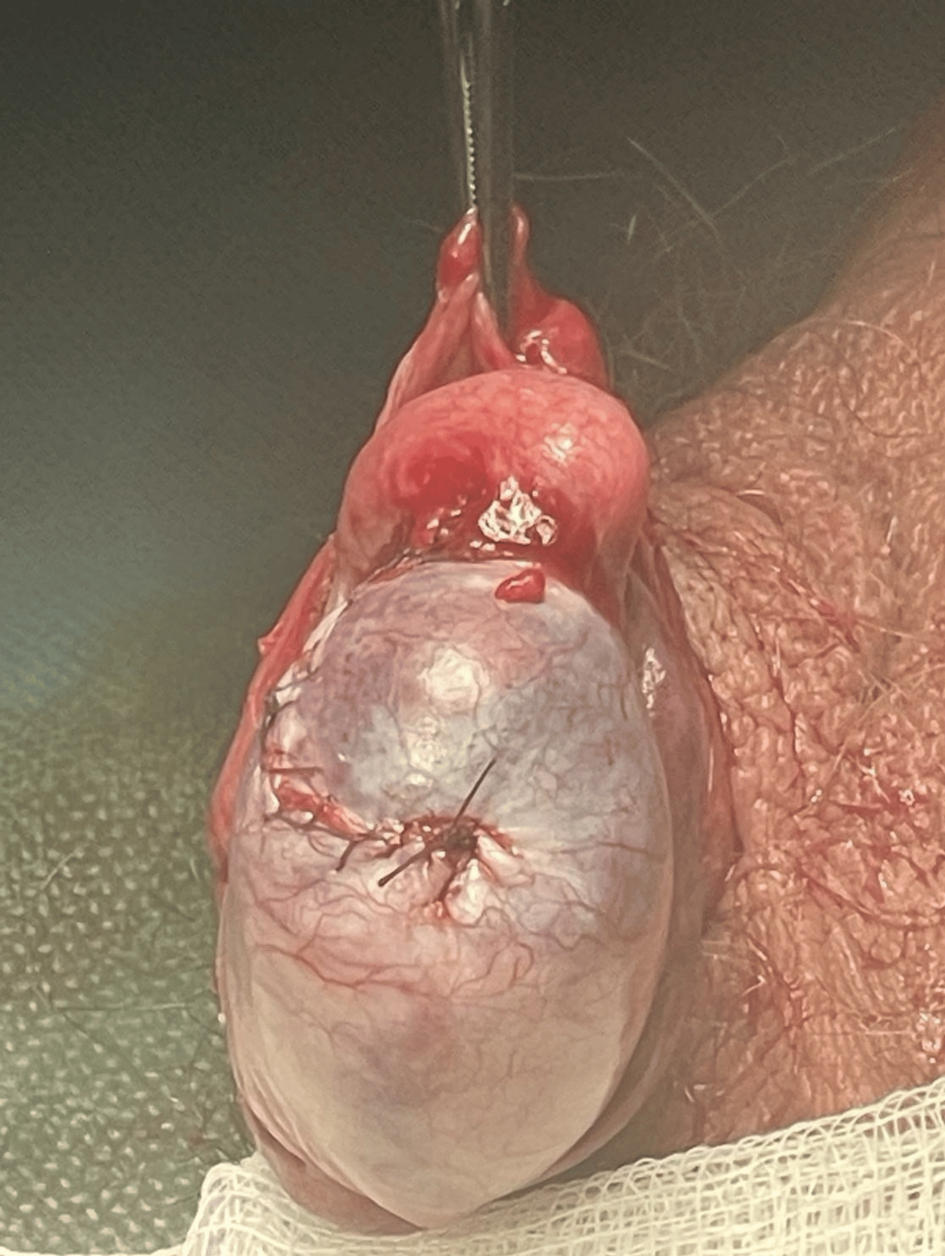
Primary closure of tunica albuginea.

The following day, the patient underwent surgical exploration. Intraoperatively, a large hematocele was evacuated. Removal of all of the necrotic forced-out seminiferous tubules was performed along with the primary suture of the tunica albuginea (Figures 5, 6).

The patient upon dismission 48 hours later was advised to avoid immediate return to physical activity for about a month. Moreover, he was encouraged to use protective cups during sporting activities with physical contact. Nonetheless, fertility testing was planned at the end of the growth.

**Figure 7:**
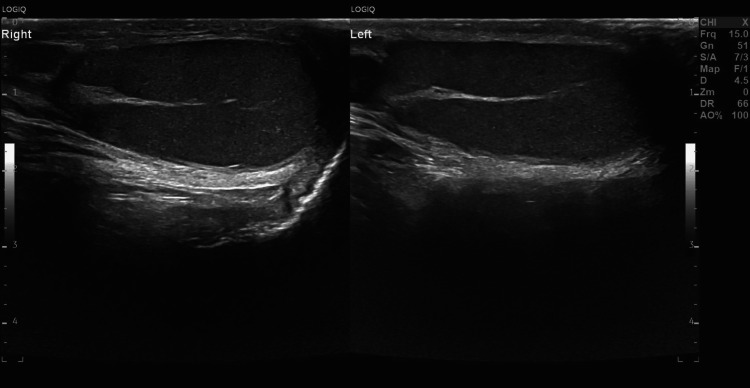
Follow-up scan. A six-month follow-up ultrasound on the sagittal plane showed a thin, echogenic tunica albuginea uniformly surrounding both testicles.

**Figure 8:**
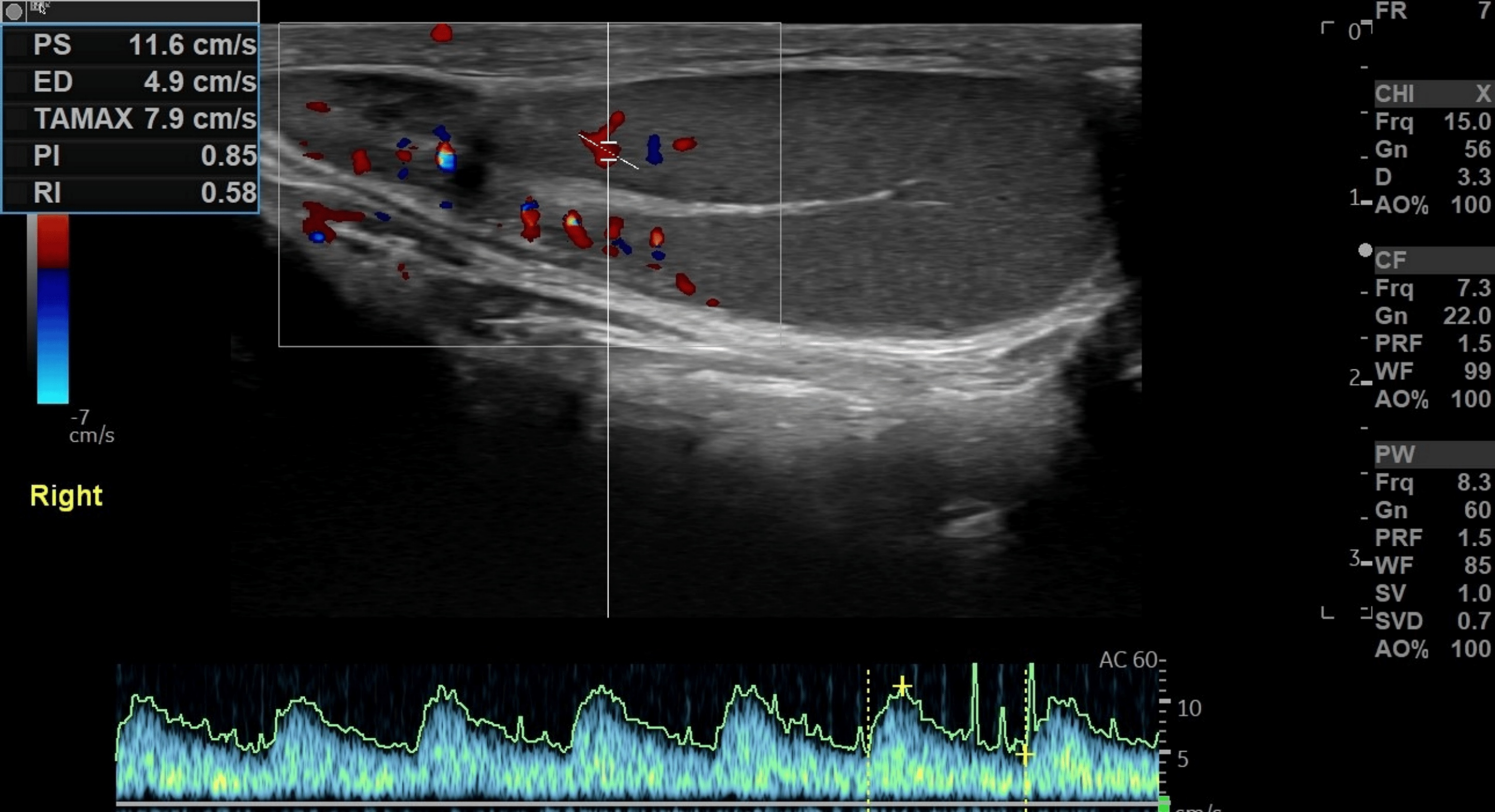
Six-month follow-up imaging evaluation. Normalization of intraparenchymal vasculature of the right testis.

Six months later, the patient had a follow-up ultrasound scrotal exam, which revealed resorption of the intratesticular hematoma and return of practically homogeneous testicular echotexture. Testicular size was within normal limits bilaterally. A thin tunica albuginea uniformly surrounding the right testis was shown (Figures 7, 8).

## Discussion

Due to anatomical reasons, scrotal trauma is an unusual emergency; however, it accounts for one of the most common causes of acute scrotal pain [2,6]. Testicular trauma, especially in young men, primarily occurs during contact athletic activities [4,5]. As seen in our case, these sports, when played without protective gear, may lead to genital trauma [4].

Scrotal injuries typically result from blunt trauma [7], which can lead to a hematoma, testicular rupture, and/or a hematocele [8]. The right testis is more susceptible to damage than the left, as it is more easily compressed against the pelvic bones or inner thigh [2,4,5]. This forceful compression can create hyperpressure within the scrotal sac, leading to a tear in the usually firm tunica albuginea that covers the mobile intra-scrotal testicle and ultimately results in the extrusion of its contents into the scrotal sac [2]. Several reports indicate that a direct force of up to 50 kg is sufficient to cause a testicular tear [2,9].

Blunt scrotal injuries may also result in testicular fractures, defined as breaks or interruptions of the testicular tissue [9]. On ultrasound, they appear as continuous hypoechoic and avascular areas within the testis. Unlike our study, these fractures can be associated with ruptures of the tunica albuginea, with only 17% of cases demonstrating a fracture line [6,9]. Intratesticular vascularity on color Doppler is another crucial indicator of the organ’s viability and aids in direct treatment planning in these cases [6].

Physical examination, in this case, revealed key clinical signs associated with testicular rupture, including a history of direct blow, acute unilateral scrotal pain, and scrotal swelling with ecchymosis. The cremaster reflex was recorded bilaterally, eliminating the possibility of concomitant testicular torsion [5].

Our patient sought medical care with an approximate delay of eight hours, jeopardizing testicular viability. Many children and young adolescents tend to initially underestimate these injuries, as demonstrated in our case, thus wasting time at the expense of prompt diagnosis and treatment.

Contemporary American Urological Association recommendations advocate for the use of ultrasonography (US) in cases of blunt scrotal trauma [4]. Testicular rupture is documented when the tunica albuginea is breached and testicular tissue is extruded into the scrotal sac. Typically, US investigations reveal contour discontinuities of the testis and distortion of parenchymal echotexture [6]. Testicular rupture ultrasound documentation based solely on tunica albuginea disruption has a reported sensitivity of 50% and specificity of 76%. However, when outline irregularity is combined with distorted testicular echotexture, the sensitivity and specificity for diagnosing testicular rupture increase to 100% and 93.5%, respectively [6].

Breaches of the tunica albuginea are usually accompanied by ischemia or absent blood flow on color Doppler sonography due to simultaneous injury to the tunica vasculosa [7]. In this context, portions of the testis lose their blood supply, forming intraparenchymal hematomas of various sizes and echotextures depending on the timing of the injury. In our case, a relatively large “avascular” echogenic area, suggestive of a hematoma, was identified at the upper pole of the testis. Nonetheless, intratesticular distortion may be present without a tunica albuginea rupture and can be treated conservatively. The concomitant irregularity of the tunica albuginea contour, detected by ultrasound, adversely affects testicular viability and requires early detection and management [6].

In our case, the patient’s blunt scrotal trauma was complicated by tunica albuginea rupture, hematomas, and hematocele, both on ultrasonography and during surgical intervention. He was initially treated conservatively with ice packs, nonsteroidal anti-inflammatory drugs, and antibiotics to allow time for hemostasis and to relieve the edematous compression on the viable testicular tissue. During surgical intervention, evacuation of the hematocele, necrotic tissue removal, and closure or fixation of the tunica albuginea should be performed accordingly [2,4,9]. Delayed exploration has been associated with a higher orchiectomy rate (45%) due to interval organization of the injury [10,11].

However, in our case, a relatively short delay of no more than 36 hours prior to surgical exploration proved beneficial. Nonetheless, poor outcomes following delayed intervention do not necessarily imply that urgent surgical reconstruction is always essential [11]. Moreover, according to Cubillos et al. [1], surgical treatment may facilitate recovery but carries the drawback of potential tissue loss due to extensive debridement. Likewise, Redmond et al. favor a more conservative approach regarding testicular trauma, as debridement may involve the removal of viable testicular tissue, and rapid closure of the albuginea may lead to testicular atrophy [11]. Constant scrotal pain or atrophy was also reported among surgically treated patients in Guichard et al.'s survey [12]. Fortunately, our patient experienced an uneventful post-surgical recovery. Additionally, most authors agree that surgical exploration within 72 hours improves the testicular salvage rate [2,13,14]. Prolonged surgical delay may significantly decrease the testicular salvage rate from 80-90% to 45-55% [4]. Consequently, the majority of clinicians strongly support that early surgical exploration is more beneficial in terms of tissue viability and faster recovery.

## Conclusions

In conclusion, testicular rupture, although rare, demands early medical attention to avoid serious complications such as infection, testicular dysfunction, and organ loss. Scrotal ultrasound is the imaging modality of choice for the diagnosis and follow-up of testicular trauma. The ultrasound findings of testicular rupture include parenchymal heterogeneity due to hematomas, discontinuity of the tunica albuginea, and hematoceles. Management options should be tailored depending on the type of injury detected. Conservative treatment can be beneficial not only for isolated intratesticular contusions or hematomas but also for the initial management of cases with additional testicular tears. Nevertheless, debridement and, most importantly, closure of the tunica albuginea are required to secure testicular preservation in cases of scrotal trauma complicated by testicular ruptures.
